# Reviewer's communication style in YouTube product-review videos: does it affect channel loyalty?

**DOI:** 10.1016/j.heliyon.2020.e04880

**Published:** 2020-09-14

**Authors:** Widia Resti Fitriani, Arif Budi Mulyono, Achmad Nizar Hidayanto, Qorib Munajat

**Affiliations:** Faculty of Computer Science, Universitas Indonesia, Depok, Indonesia

**Keywords:** Product review, Communication styles, Channel loyalty, Motivation theory, Indonesia, E-commerce, Information systems, Internet-based information systems, Information technology, Technology management, Technology adoption

## Abstract

Product-review videos can be a promising marketing method. The popularity of using videos as a medium for product reviews is evidenced by the number of channels that are used to provide product reviews on multiple platforms. Reviewers can use various strategies to attract wider audiences and make these audiences loyal to their channels. This study analyzes the effect of the reviewer's communication style on the audience's loyalty to the channels that provide product reviews, especially on the YouTube platform. Communication-style analysis is associated with hedonic and utilitarian motivation theory, which can be used to explain communication style effect on channel loyalty. This study uses a quantitative approach using questionnaire to obtain data. The data in this study are analyzed using covariance-based structural equation modeling in AMOS 21.0. The results show that communication styles (social-oriented and task-oriented), perceived transparency, perceived enjoyment, credibility, and channel engagement affect channel loyalty. Meanwhile, the informativeness factor does not affect channel loyalty. This paper will also discuss the theoretical and practical implications of the study.

## 1 Introduction

The popularity of computer and gadget usage has increased dramatically eliminating the restrictions on people's ability to interact virtually [1]. The ease of virtual interaction allows people to search and exchange information easily. Finding and exchanging information about products is one such activity that is undertaken. Information about products that are provided by product users on the Internet is usually called online reviews [2]. Online reviews can be delivered in a variety of forms, namely text, audio, and video. Reviews in text form—for example, a blog, review column, or a form of rating—are the most commonly found type. Reviews in audio form are done via radio broadcasts, podcasts, or other means, while reviews in video form are commonly television broadcasts. In the online context, the use of video as a medium for product reviews is a new and developing trend.

Online reviews using videos are predicted to increase in popularity in the next few years. In 2019, online videos were estimated to account for 80% of all traffic on the Web [3]. As many as 90% of customers stated that a video about a product could help them make a purchase decision about it [3]. The number of videos on the YouTube platform is growing by 100% every year; it is the largest platform in the online video field [3]. As many as 64% of customers are more interested in buying a product after watching a video about it [3]. Several types of social media platform, including YouTube and Instagram, are used to provide reviews via video format, for example, “Ask the Android Guy”, “Unbox Therapy”, and “MobileTechReview” [4, 5].

Using videos, reviewers can convey a great deal of information through language both verbally and nonverbally. According to the previous studies, several factors can influence user loyalty in the context of online videos on YouTube. These factors include awareness, exploration, and relationship [6]; relevance and popularity [7]; access to information using motivational theories [8]; and audience perception, similarity, expertise, and likability [9].

Communication style has not been discussed among the various factors that have been studied previously. Communication style is determined based on overall character of an individual communicative act [10]. Communication style is also an individual tendency to communicate using specific patterns and rules [11]. The communication-style factor is essential because it influences sales performance [12]. In addition, the influence of this factor is likely to be strong or dominant and to affect audience behavior. Therefore, the aim of this study is to determine how communication styles can influence the audience intention to watch online reviews again and recommend the videos to others (loyalty).

Many theories explain the grouping of communication methods. One study identifies two types of communication methods—namely, social-oriented and task-oriented [13]. Based on an analysis of online review videos, this study will examine the relationship between communication style and user behavior. This study will analyze the loyalty behavior of the audience toward channels that conduct online reviews. Loyalty is the tendency of the audience to remain loyal and willing to rewatch channels on which online reviews are conducted [14]. Loyalty is also the tendency of the audience to watch and recommend the videos on the channel [15].

This study uses hedonic and utilitarian motivational theories to explain the relationship between communication styles and user behavior on video reviews on YouTube. These theories are often used to explain user behavior in many domains, including the use of technology [16]. Therefore, by combining theories about communication and motivation, this research is expected to explain the impact of the communication styles of YouTube product reviewers on channel loyalty.

This research is expected to make theoretical and practical implications. Theoretically, it will help enrich the literature and knowledge related to video review analysis, specifically on the topic of communication styles and their impact on audience engagement and loyalty. Practically, for individuals who have review channels on YouTube, this study can describe the kind of communication styles they should use when reviewing products to help make their audiences feel engaged with and loyal to their channels.

## 2 Literature study

This section explains the theories of communication styles: hedonic and utilitarian motivation, engagement, and loyalty. We also discuss previous research, which becomes the primary reference in this study.

### 2.1 Communication styles

Communication styles refer to the tendency of individuals to communicate using specific patterns or rules [11]. Communication styles are all the characteristics of individual communicative action [10]. Communication styles can be used in daily life activities, such as teaching and learning [17], buying and selling [18], and information sharing. Communication styles are not only used when offline or face to face. With the help of technology, communication styles can also be used online. One technology platform that enables online communication is YouTube. Communication on YouTube allows two-way interaction during live streaming. However, this study discusses the standard video format that allows one-way communication only.

There are several types of communication styles—namely, linear vs. circular communication, direct vs. indirect communication, formal vs. informal communication, low-context vs. high-context communication, attached (emotive) vs. detached (non-emotive) communication, idea-focused (intellectual confrontation vs. person-focused relational confrontation) communication, task-oriented vs. social-oriented vs. self-oriented communication, and concrete vs. abstract communication [19]. Communication styles can also be divided into five types—namely, assertive, aggressive, passive–aggressive, submissive, and manipulative [20]. Another theory that describes the style of communication is communication accommodation theory (CAT), which explains and predicts how and why people shift their languages, dialects, and accents when interacting with each other and model how others in an interaction perceive, evaluate, and respond to them [21]. CAT is one theory that can be used to avoid conflicts in communication [21]. For this context, the study will discuss social-oriented, task-oriented, and self-oriented communication only [13].

A social-oriented communication style is a communication style that prioritizes cooperation and tends to avoid conflict [22]. A social-oriented communication style also aims to personalize and establish good relationships between individuals [11]. This communication-style orientation is generally friendly, supportive, respectful, dependable, and agreeable [22]. Individuals who use this style of communication are more interested in building good relationships with others, and they pay attention to comfort when interacting. Individuals who prefer this style of communication believe that personalization is an essential part of the interaction process. In the context of sales, a salesperson will listen to the buyer's opinion and feel happy to interact with the buyer. Buyers who feel cared for will feel comfortable and valued, enabling them to be more engaged with the sellers. This engagement will have a positive impact, which can be an intention to buy, to recommend, to be loyal, and other positive outcomes. However, in this communication style, a considerable amount of time to build a good relationship is lacking.

The task-oriented style is a communication style that is oriented toward achieving specific goals or completing tasks. This communication style focuses on the efficiency of the task both in terms of time and cost [11]. Individuals who like this style of communication tend to follow mechanistic procedures and standard rules in their approaches to others [18]. In the context of sales, the seller who uses this style of communication tends to focus more on achieving sales targets. The seller has a clear purpose and a structured target. This communication style has a positive impact on the efficiency of the sales process in a short time. However, there are drawbacks to using this style of communication—for instance, an excessive focus on sales will reduce the focus on buyers.

The self-oriented style is a style of communication that places more importance on oneself than on other people. The concepts of pleasing oneself and having ambition tend to dominate this style of interaction [18]. In the context of interactions between salespersons and customers, salespersons who use this style of communication tend to be preoccupied with themselves when interacting and to lack empathy for customers [11]. Based on research conducted by [11], salespersons who are self-oriented will produce the fewest sales variants; therefore, the style will hinder sales and is not suitable for salespersons. The use of a self-oriented communication style in an review on YouTube is not suitable because it tends to entail an excessive orientation toward oneself, which will create a negative evaluation effect, especially with respect to the audience's perception of the reviewer.

Several studies have analyzed this communication style in various fields, including retail [13]. Research conducted by [13] analyzed that the interactions with salespersons in retail stores contribute to building trust and profit. In most impersonal interactions on the Internet, interactive animated characters or avatars play the social role of online sales assistants. They must have a strong social presence and have the potential to replace some direct interaction. This paper examined whether avatars with a task- or social-oriented communication style contribute to user trust and patronage intentions. Further testing explored the moderating effect of the type of product/service. The study included online experiments and surveys involving 636 participants, who were mainly from North America, Oceania, and Europe and were randomly allocated to various treatments. Task-oriented communication contributes to trust, which then contributes to patronage intentions, especially the intention to search for goods/services. Socially oriented communication also contributes directly to trust and patronage intentions, especially for goods/services that are trusted.

Another study [23] explored the impact of similarity in communication style on intelligent advisory among users in the health domain. Referring to the similarity–attraction theory, a research framework is proposed to investigate this relationship. This paper used a mixed-methods approach, —namely, survey, experiment, and interviews. The results of the study show that when the intelligent advisory communication style matches the communication style of the user, the user will be more involved with the system; this engagement results in better perceived transparency, enjoyment, informativeness, and credibility during the interaction process. Overall, with regard to creating an atmosphere of social presence and fostering an intention to reuse, the hedonic aspect of user communication-style equality is more important than the utilitarian aspect of its usefulness.

### 2.2 Motivation theory

One's communication style can affect other people's perceptions of the information that is conveyed. This perception can build a person's motivation to carry out an activity. Motivation is a process that influences an individual to perform a specific behavior [24]. Motivation is the reason underlying an action that is performed by an individual. A person is said to have a high motivation if he or she has very strong reasons for doing what he or she wants. In the context of sales, two main motivations drive purchases—namely, hedonic and utilitarian motivation [25].

Hedonic motivation is a motivational impulse that is influenced by the pleasure and pain that an individual derives from doing or staying away from a particular activity [26]. This concept is related to the classic motivational principle that people like pleasure and tend to avoid pain [26]. The hedonic aspect makes a significant contribution to individual feelings toward sociable, warm, personal, or intimate interactions [23]. Both pleasure and pain are the results of certain emotion-related behaviors, such as love, hate, fear, and joy [27]. According to the hedonic motivation theory, the emotional experience can be considered a measuring needle that has a scale from pain to pleasure; so, it is as if the primary motivation of the individual is to always keep the measuring needle as close as possible to pleasure [28]. Hedonic motivation drives the desire to experience something that is personally pleasing.

Utilitarian motivation is the desire to obtain products that can be used based on their usefulness to achieve a goal [29]. The utilitarian motivation can be illustrated as follows: When an individual needs to refuel his or her car, the person will buy gasoline. In the context of purchasing goods, utilitarian motivation views efficiency, timeliness, and minimal irritation when purchasing goods is very important for an individual [25]. Therefore, products that are commonly purchased based on utilitarian motivation are price sensitive [29].

### 2.3 Channel engagement and loyalty

In the context of buying and selling, customer engagement is a business communication relationship between external stakeholders (consumers) and organizations (companies or brands) that is maintained through various existing channels [30]. The relationship can be a reaction, interaction, and customer experience that occurs offline and online. Customer engagement can also be defined as a psychological state that encourages more frequent interactions with focal objects, such as brands [31]. Customer engagement is a long-term relationship that arises in terms of emotional and utilitarian motivational encouragement [31]. In this study, engagement is applied to the online realm, which is a channel that provides reviews on the YouTube platform. Channel engagement illustrates the close relationship between the viewer and the YouTube channel.

Meanwhile, customer loyalty is a positive relationship between the customer and the organization (company or brand) that causes the customer to be satisfied and happy with goods or services that meet his or her expectation to foster an intention to buy or use the brand again [32]. Customer loyalty can also be defined as a tendency for customers to consistently prefer an item or service made by a particular company or brand [33]. In this study, loyalty is applied to the online realm, which is a channel that provides reviews on the YouTube platform. The loyalty channel illustrates the tendency of viewers to watch videos from the channel again.

A study by [34] examined whether back-channel communication during major sporting events has a positive impact on loyalty to sports channels. A survey with 500 samples taken randomly from national panel data in South Korea was conducted right after the 2014 Sochi Olympics. Based on fundamental theories on Web interactivity, emotional attachment, and company–consumer identification, three levels of engagement were proposed on social TV. Using confirmatory factor analysis, tripartite dimensions of social media engagement—namely, functional, emotional, and communal—were produced to the audience on social TV. Data analysis using structural equation modeling (SEM) showed that functional engagement has a direct impact on social presence and that communal engagement has a direct impact on channel loyalty. In addition, the impact of emotional engagement and social presence on channel loyalty is mediated by the channel commitment.

### 2.4 The stimulus–organism–response (SOR) model

The stimulus–organism–response (SOR) model is a theoretical framework that can replicate a particular behavior of an individual when he or she is receiving a stimulus from a communication process [35]. This model assumes that verbal and nonverbal communication will stimulate individuals to respond [36]. This model shows that communication is a process of action and reaction. The SOR model has three elements—namely, stimulus, organism, and response.

The stimulus is an influence that moves an individual to act [37]. In the context of brand communities on social media, the stimulus is the motivation to participate in communities that affect the internal circumstances of customers [38]. In the e-commerce context, the stimulus is the total number of cues that can be seen or heard by customers [37]. In this study, the communication style of the reviewer on YouTube is a trigger to become a particular stimulus.

The organism, the second component of the SOR model, refers to the cognitive and affective aspects of the message recipient in processing the stimulus and response given [39]. In the affective aspect, the recipient processes the stimulus and response in terms of feelings and emotions [37]. In the cognitive aspect, the message recipient processes the stimulus and response logically. Both of these aspects help process the stimulus so that it becomes meaningful information that can support decision-making regarding the response behavior based on what the individual wants to do [39]. In this study, what constitutes an organism is the audience watching the video review.

The response, the third component of the SOR model, refers to the consequences of the behavior that is carried out by the recipient of the message toward the given stimulus [40]. The responses of stimulus recipients can be positive or negative behavior [37]. In the context of buying and selling, positive responses can be reflected in the form of purchases, intentions to recommend, and other reactions. The negative response can be reflected in the form of containment intention to buy, negative communication, and other reactions. This study will examine the effect of communication-style stimulus on the response of the audience watching the review, as well as its effect on engagement and loyalty channels.

## 3 Research model and hypotheses

This section will explain the development of the research model and the hypotheses used in this study.

### 3.1 Research model

**Figure 1:**
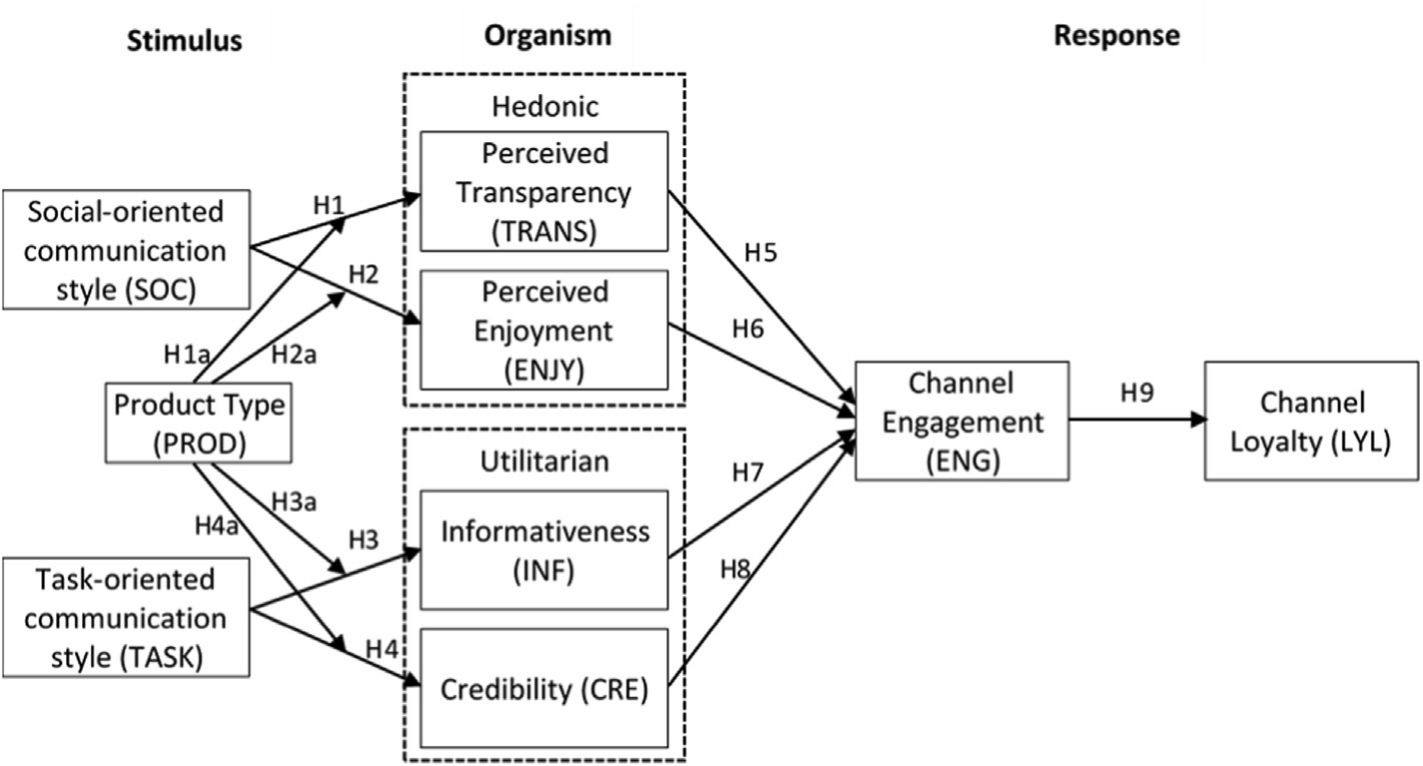
Research model.

In this research, we combine several approaches to develop the research models. This study refers to the research model by [34] as a theoretical framework for forming engagement channels and loyalty channels for product reviews on YouTube. In this study, the formation of engagement channels and loyalty channels uses an SOR model. In the first model, stimulus, we use the reference variable from research by [13]—namely, the social-oriented and task-oriented communication style. In the second model, organism, we use a motivational perspective from the hedonic and utilitarian concepts to evaluate the audiences of product reviews on the YouTube platform. In this second model, we refer to research by [23]. The hedonic motivation perspective consists of perceived transparency and perceived enjoyment variables, while utilitarian motivation consists of informativeness and credibility variables. In the third model, response, we use the reference variable from the study of [34] by adopting channel engagement and channel loyalty variables. The research model can be seen in Figure 1.

### 3.2 Hypotheses development

The social-oriented communication style will help an individual understand the information without an in-depth explanation because the delivery tends to be friendly [41]. Besides, individuals will feel more comfortable; therefore, they can develop increased feelings of transparency and understanding regarding what is conveyed by other individuals [23]. In the context of online video reviews, a social-oriented communication style also influences perceived transparency. Therefore, the following hypothesis is proposed:

Hypothesis 1 (H1) A social-oriented communication style influences perceived transparency.

Enjoyment is an affective feeling that refers to the extent to which an activity is considered plasurable [42]. Enjoyment is one of the main factors that contribute to consumer behavior patterns [43]. Although enjoyment is often regarded by some researchers as a parent concept for engagement, some other researchers view these two concepts as having different focus [44]. A social-oriented style of communicating with an individual is expected to have a direct effect on the creation of a pleasant interaction; therefore, it can facilitate communication and result in a low likelihood of conflict [23]. Previous research also shows that the social-oriented communication style has a significant positive impact on perceived enjoyment [45]. In the context of video reviews, the reviewer's social-oriented communication style will have an influence on audience enjoyment. Therefore, the following hypothesis is proposed:

Hypothesis 2 (H2) A social-oriented communication style influences perceived enjoyment.

Informativeness is an individual's perception that other individuals can convey relevant information effectively [46]. Informativeness also describes the individuals' feelings that they are being informed about a particular product or service, along with their experiences associated with the use of this product or service [47]. Individuals who use a task-oriented communication style can effectively provide a great deal of information in a short time [11]. Interaction using this communication style tends to focus on what is to be conveyed to minimize distractions and deliver complete information [13]. In the context of video reviews, a task-oriented communication style influences informativeness. Therefore, we propose the following hypothesis:

Hypothesis 3 (H3) A task-oriented communication style influences informativeness.

Credibility can be defined as the extent to which individuals can trust other individuals [48]. In the context of this study, the definition becomes the extent to which the audience can trust the reviewer. A person who has a task-oriented communication style tends to focus on what he or she wants to convey so that the communication appears more professional and reliable [13]. Consequently, the following hypothesis is proposed:

Hypothesis 4 (H4) A task-oriented communication style influences credibility.

Perceived transparency is how communication or behavior in individuals can be conveyed to other individuals so that it is easy to see or understand what is conveyed or done [49]. Transparency is also a subjective perspective on how easy or difficult it is for other individuals to understand what is conveyed [50, 51]. Meanwhile, engagement is a conceptualization of full attention that requires cognitive effort and the in-depth processing of new information [44]. The easier it is for individuals to understand what is conveyed, the easier it will be for them to give their full attention to become engaged with what is conveyed. Research by [52] showed that transparency would affect channel engagement. Therefore, the following hypothesis is proposed:

Hypothesis 5 (H5) Perceived transparency influences channel engagement.

Perceived enjoyment is a positive reward in the form of pleasure that intrinsically arises when an individual carries out a particular activity [42]. If someone feels happy when watching online video reviews on a particular channel, this person will be easily engaged with this channel. Research by [53] showed that perceived enjoyment affected channel loyalty. Audiences who are happy, absorbed, and interested in seeing video reviews will potentially be engaged with the channel. Therefore, the following hypothesis is proposed:

Hypothesis 6 (H6) Perceived enjoyment influences channel engagement.

In the context of online reviews, informativeness refers to the completeness of the information that the reviewer can give to the audience [54]. Informativeness can measure the extent to which information or explanations from reviewers can be conveyed to audiences [55]. Informativeness is a combination of three factors—namely, information, linguistic meaning, and context—that allow the audience to draw conclusions and recognize what is communicated [54]. Pragmatically, the study of language states that the audience needs to expect the message to be informative, trustworthy, relevant, concise, and clear to make them understand the message from the speaker. Research by [56] in the field of mobile advertising showed that informativeness influenced engagement. Research conducted by [57] also showed that perceived informativeness influenced engagement. Therefore, the following hypothesis is proposed:

Hypothesis 7 (H7) Informativeness influences channel engagement.

Engagement is the natural closeness of the individual to the experience of interaction [58]. Engagement is also closeness that includes cognitive, emotional, and behavioral perspectives [59]. In the context of marketing, credibility is identified as the antecedents of consumer engagement [60]. In the online review context, if the reviewer provides information in a neutral, reliable, and trustworthy manner, it can potentially increase the audience's interest in being loyal—for example, by giving likes/dislikes, commenting, or fostering the intention to watch videos on the particular channel more often. Therefore, the following hypothesis is proposed:

Hypothesis 8 (H8) Credibility influences channel engagement.

Engagement can be an essential predictor of loyalty building [61]. Another body of research states that engagement with social media has a direct impact on loyalty [58]. A product-review audience who often gives likes/dislikes or makes comments can potentially become oyal to the channel. In the context of video reviews, engagement with the channel is expected to build the audience's loyalty to the channel. Therefore, the following hypothesis is proposed:

Hypothesis 9 (H9) Channel engagement influences channel loyalty.

## 4 Research methodology

This research was conducted in eight stages consisting of the problem formulation, literature review, research model formulation, questionnaire preparation, readability test, data collection, data processing and analysis, and study conclusions and implications. This section describes the methodology that was employed for data collection, instrument development, and data analysis.

### 4.1 Data collection and sample

This study adopted a quantitative research approach, using a questionnaire as the primary means of data collection. We used a non-probability sampling method to select the respondents. We employed the convenience sampling technique, whereby samples are selected from the population only because they are conveniently available to the researchers. Although such samples do not represent the entire population, we chose them because they are easy to recruit. The questionnaires were distributed online using the Google Form service. The online questionnaires were distributed via social media, such as Instagram, Facebook, and Twitter, as well as via instant-messaging media, such as Line and WhatsApp. In this study, the target respondents were people who had seen a product review on the YouTube platform. Data retrieval lasted for one month: from April 1, 2018 to May 1, 2018. After one month, we stopped the data collection process; we obtained 680 respondents. However, some incomplete and non-analyzable data remained and needed to be eliminated. After these data were removed, there were 638 respondents. The amount of data met the rule for a representative sample size that can be analyzed using the SEM method—that is, 10 times the number of instruments used [62]. Table 1 shows a summary of the demographic data.

**Table 1:** Respondent demographics.

Demographic Variable		Total	Percentage
Gender	Male	308	48%
	Female	330	52%
*Age*	<17 years old	137	21.5%
	17–23 years old	438	68.5%
	24–30 years old	24	4%
	31–40 years old	11	2%
	>40 years old	28	4%
*Education*	Elementary/middle/high school or equivalent	495	77.5%
	Diploma	14	2.2%
	Bachelor	110	17.2%
	Magister	12	2%
	Doctoral	1	0.1%
	Other	6	1%
*Current Occupation*	Student	555	87%
	Civil servant	15	2.25%
	Private employee	31	5%
	Entrepreneur	15	2.25%
	Other	22	3.5%
*Type of Product Review*	Gadget	183	28.9%
	Automotive	54	8.5%
	Game	90	14.1%
	Software	6	1%
	Foods & beverages	56	8.7%
	Fashion	18	3%
	Cosmetics	200	31%
	Tourism	20	3.1%
	Other	11	1.7%

### 4.2 Instrument development

The instrument development was conducted after the formulation of the research model. All research instruments were developed by identifying and selecting indicators from previous studies. The indicators from the previous studies were adapted and modified to suit the context of the study. The adaptation and modification of the previous indicators were carried out because their reliability and validity had been tested. Furthermore, after all the indicators were complete for each variable in the model, the measurement scale was determined. This study used a Likert measurement scale consisting of five options: strongly disagree, disagree, neutral, agree, and strongly agree. A five-point Likert scale was employed, as it has been most recommended by the researchers because it reduces respondents’ frustration level and increases response rate and response quality.

The final questionnaire consisted of 36 questions based on eight different constructs of the proposed research model: social-oriented communication style (six items), task-oriented communication style (three items), perceived transparency (five items), perceived enjoyment (three items), informativeness (five items), credibility (four items), channel engagement (six items), and channel loyalty (four items). The validation and readability test of the questionnaire was conducted by researchers and experts.

### 4.3 Data analysis

This study used the covariance-based SEM (CB-SEM) method to process and analyze the data on AMOS 21.0. Before the analysis, the data were cleaned by removing the outliers. The analysis phase started with the specification of the model, identification and estimation of the model, measurement model test, structural model test, and model interpretation.

The path diagram represents the research model along with the variables and indicators that were defined. The path diagram had eight variables and 36 indicators. Among these variables, there were two exogenous and six endogenous variables. Social-oriented and task-oriented communication styles were the exogenous variables. The other variables—namely, perceived transparency, perceived enjoyment, informativeness, credibility, engagement channels, and loyalty channels—were endogenous. After the path diagram was created, we identified the model by looking at the degree-of-freedom (DOF) value to determine whether the model had a sufficient solution [63]. The DOF value on the path diagram was positive (DOF = 584), indicating that this research model had many solutions (overidentified). In addition, the path diagram had a chi-square value of 1718.938.

The number of respondents obtained was 638. This amount of data met the representative sample-size rules for the SEM method—that is, the data were 10 times the number of instruments used [62]. The SEM method requires the data that are collected to be normally distributed. A data normality test can be done by checking the univariate for each indicator and multivariate for all variables [63]. The normality test aims to determine the skewness and kurtosis value, which must be in the range of -2.58 to +2.58. Outliers are data that are far above or below the average of the available data [63]. Outliers cause data that are not normally distributed; thus, elimination of the data needs to be carried out. We conducted two iterations of the outlier elimination process, with a total of 60 respondents eliminated. The total number of respondents also changed from the initial 638 to 578. After the deletion of the outliers, the data were still not normally distributed. Hence, we employed a bootstrapping process by resampling the data to 600 with a confidence level of 95%.

## 5 Result

This section discusses the analysis results of the research model using the SEM method. This section consists of two main results—that is, the assessment results of the measurement model and the structural model.

### 5.1 Assessment of measurement model

The aim of the measurement model test is to determine the relationship and closeness between the variables and their constituent indicators and how well they can explain the variables [63]. The measurement test consisted of several stages, which included reliability indicators and internal consistency reliability tests. In addition, the stages of validity testing included convergent validity, discriminant validity, and goodness-of-fit (GOF) tests.

The reliability test is carried out to determine the level of consistency of a research-measuring instrument [64]. In this test, two things were seen—namely, the value of construct reliability or composite reliability (CR) should be ≥0.7, and Cronbach's alpha (CA) should be ≥0.7 [63]. The validity test is used to measure the accuracy of the research indicators [63]. The validity test is divided into two—namely, the convergent validity test and the discriminant validity test. The convergent validity test is conducted to determine the relationship between the same variable constructs in the research model [63]. In this test, two things were seen—namely, the loading factor value should be >0.7, and the average variance extracted (AVE) should be >0.5 [63]. In this test, several indicators had factor loadings of less than 0.7—namely, indicator CRE3, ENG1, ENG2, ENG3, LYL4, SOC1, SOC3, SOC4, and TRANS4—and needed to be deleted. Table 2 shows the calculation results of the factor loading, CA, CR, and AVE values.

**Table 2:** Factor loadings, AVE, CR, and CA.

Variable	Indicator	Factor Loadings	AVE	CR	CA
CRE	CRE1	0.845	0.680	0.864	0.861
	CRE2	0.854			
	CRE4	0.773			
ENG	ENG4	0.82	0.701	0.876	0.874
	ENG5	0.844			
	ENG6	0.848			
ENJY	ENJY1	0.92	0.801	0.924	0.922
	ENJY2	0.911			
	ENJY3	0.853			
INF	INF1	0.766	0.629	0.895	0.894
	INF2	0.81			
	INF3	0.808			
	INF4	0.819			
	INF5	0.762			
LYL	LYL1	0.835	0.608	0.822	0.818
	LYL2	0.794			
	LYL3	0.704			
SOC	SOC2	0.982	0.815	0.928	0.806
	SOC5	0.711			
	SOC6	0.987			
TASK	TASK1	0.987	0.975	0.991	0.721
	TASK2	0.988			
	TASK3	0.987			
TRANS	TRANS1	0.844	0.655	0.883	0.997
	TRANS2	0.852			
	TRANS3	0.758			
	TRANS5	0.779			

The discriminant validity test is carried out to determine the relationship between different constructs of variables in the research model [63]. In this test, two things were seen—namely, the comparison value of the AVE roots with the correlation between the constructs (latent variables) and cross-loading [63]. Table 3 shows the square root of the AVE, which indicated that the root values of the AVE were bigger than the correlations between the other factors. Table 4 shows the cross-loading value.

**Table 3:** Square root of AVE.

	CRE	ENG	ENJY	INF	LYL	SOC	TASK	TRANS
CRE	**0.825**							
ENG	0.571	**0.837**						
ENJY	0.672	0.686	**0.895**					
INF	0.753	0.527	0.662	**0.793**				
LYL	0.525	0.569	0.468	0.437	**0.780**			
SOC	0.562	0.510	0.672	0.619	0.426	**0.903**		
TASK	0.594	0.539	0.612	0.662	0.400	0.560	**0.987**	
TRANS	0.712	0.587	0.703	0.737	0.467	0.665	0.696	**0.809**

Based on the results, we can conclude that internal consistency, reliability, convergent validity, and discriminant validity were met. For measurement model testing, GOF testing is performed using absolute fit indices, incremental fit indices, and parsimony fit indices to determine how well the model fits the research data obtained. Several commonly used GOF indices and their values are the chi-square (0.071), CMIN/df (1.129), GOF index (GFI = 0.962), root mean square error of approximation (RMSEA = 0.015), comparative fit index (CFI = 0.997), normed fit index (NFI = 0.972), and adjusted z of fit index (AFGI = 0.948). The values indicated that the GOF testing results met the criteria (good fit) and fit the sample data.

### 5.2 Assessment of structural model

Structural model testing consists of several stages—namely, the structural GOF test, hypothesis testing, direct effect closeness test, squared multiple correlations, and the final research model. In the structural test, a moderating GOF test is performed to determine whether a variable can moderate the relationship between variables. In this test, there were additional moderation variables—namely, product types. The GOF test showed that the values of the GFI (>0.90), RMSEA (0.07), CFI (>0.90), NFI (>0.90), and AGFI (>0.80) met the criteria.

The next step was to test the hypothesis using two direction (two-tailed) schema. This was done by testing the p-value that had a significance of 5% in regression weight. If the value of p < 0.05, the research hypothesis will be accepted, but if the value of p ≥ 0.05, the research hypothesis will be rejected [63]. Table 4 shows the results of hypothesis testing, while Tables 5 and 6 show the results of hypothesis testing for the moderating variable.

**Table 4:** Hypotheses testing results.

Hypothesis	Parameter	Estimate	p	Result
H1	SOC → TRANS	0.444	0.003	Accepted
H2	SOC → ENJY	0.562	0.003	Accepted
H3	TASK → INF	0.378	0.003	Accepted
H4	TASK → CRE	0.421	0.003	Accepted
H5	TRANS → ENG	0.156	0.03	Accepted
H6	ENJY → ENG	0.465	0.004	Accepted
H7	INF → ENG	-0.015	0.918	Rejected
H8	CRE → ENG	0.258	0.004	Accepted
H9	ENG → LYL	1.025	0.005	Accepted

**Table 5:** Hypotheses testing for moderating variable (search goods).

Hypothesis	Parameter	Estimate	p	Result
H1a	SOC → TRANS	0.395	0.004	Accepted
H2a	SOC → ENJY	0.5	0.007	Accepted
H3a	TASK → INF	0.888	0.003	Accepted
H4a	TASK → CRE	1.015	0.004	Accepted

**Table 6:** Hypotheses testing for moderating variable (Experience goods).

Hypothesis	Parameter	Estimate	p	Result
H1a	SOC → TRANS	0.523	0.003	Accepted
H2a	SOC → ENJY	0.664	0.004	Accepted
H3a	TASK → INF	0.948	0.003	Accepted
H4a	TASK → CRE	1.122	0.005	Accepted

**Figure 2:**
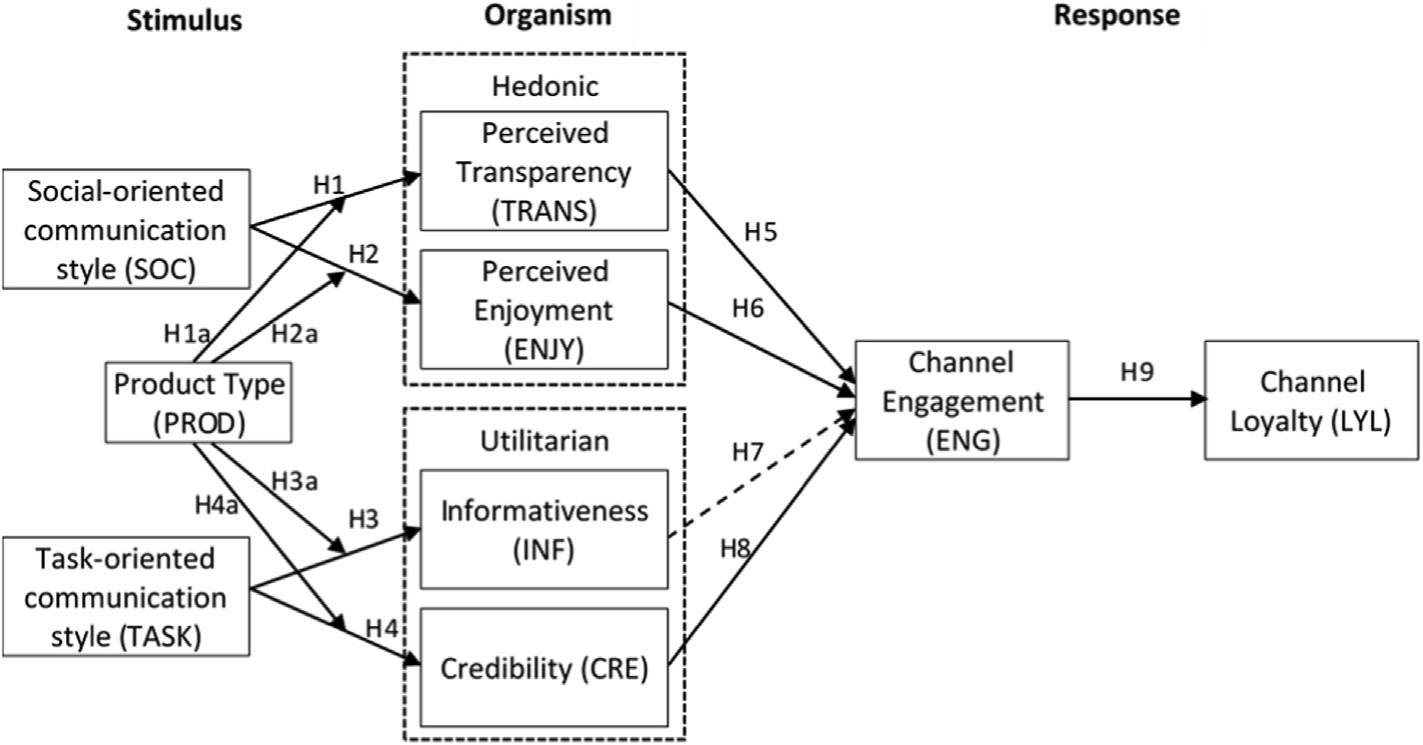
Final research model.

According to the summary of hypothesis testing in Table 4, among the nine hypotheses proposed in this study, eight are accepted, and one (H7) is not accepted. In addition, according to Tables 5 and 6, both types of products (search goods and experience goods) moderate hypotheses 1 through 4. Figure 2 shows the final research model based on the results of hypotheses testing.

## 6 Discussion

This study aimed to analyze the influence of communication styles on loyalty channels for YouTube product reviews. Based on the analysis results, social-oriented and task-oriented communication styles have an influence on channel loyalty through hedonic motivation (perceived transparency and perceived enjoyment), utilitarian motivation (credibility), and channel engagement. This study also determined that type of product (search and experience goods) moderates the relationship between social-oriented communication styles with perceived transparency and perceived enjoyment, as well as the relationship between task-oriented communication style with informativeness and credibility. Based on the results of the analysis, it can be stated that both social-oriented and task-oriented communication styles foster loyalty to the review channel on YouTube. The results of the analysis are in line with research by [13], who state that communication style influences loyalty.

This study confirms that the social-oriented communication style influences perceived transparency and perceived enjoyment. These results support research conducted by [23, 41, 45]. In the context of online video reviews, a social-oriented communication style prioritizes delivery that tends to be friendly so that it will help the audience understand the information without an in-depth explanation. In addition, this type of communication can create a pleasant interaction that will make the audience feel comfortable, facilitate its understanding of what is conveyed by the reviewer, and reduce the possibility of conflict.

This study also found that the task-oriented communication style influences informativeness and credibility. These results support research conducted by [11, 13]. Reviewers who use the task-oriented communication style can effectively provide a great deal of information to the audience in a short time. Interaction with this communication style tends to focus on what is to be conveyed in order to minimize distraction and ensure that the information is conveyed completely. Moreover, reviewers who use this communication model appear more professional and reliable because reviewers focus on the content of the information they want to convey.

The results of the study show that hedonic motivation (perceived transparency) influences channel engagement. These results indicate that an easily understood explanation by a reviewer will affect audience engagement with the channel. This result is in line with research by [52], which states that transparency affects channel engagement. The calculation results also show that the hedonic motivation (perceived enjoyment) influence channel engagement. This result is in line with research conducted by [53]. This means that if the channel is fun, audience engagement will be affected favorably.

In addition, utilitarian motivation (credibility) influences channel engagement. With this influence, if the product-review channel provides information that is true, trustworthy, and reliable, audience engagement with the channel will also be affected. This result is in line with research by [60, 65], which states that credibility is an antecedent of engagement. However, based on the results of this study, utilitarian motivation (informativeness) was found not to affect channel engagement. This result can occur because the indicators of informativeness cannot cover the perceptions of the information needs of each user; therefore, other indicators are needed to meet these needs.

Finally, the calculation results also show that channel engagement affects channel loyalty. If the audience is focused while watching the video review, this can be an indicator of loyalty to a good channel. This result is in line with research by [61], which states that engagement can be an important predictor of loyalty building. Based on the overall results, the hedonic and utilitarian values influence engagement and loyalty channels. From the results of the analysis, it can be stated that good hedonic and utilitarian values will foster audience engagement and loyalty toward the channel that review products on YouTube. These results are in line with research by [52, 53, 61, 65].

## 7 Conclusion

This research was conducted to analyze the relationship between communication styles used during product-review videos on YouTube to channel loyalty. The method used in this study was a quantitative approach, which entailed distributing questionnaires to respondents who have watched product-review videos on the YouTube platform. The number of respondents obtained from the distribution of questionnaires was 680. The distribution of the questionnaires, data processing, and analysis were performed using the CB-SEM method. Based on the analysis, the factors that influence channel loyalty based on online product reviews on YouTube are social-oriented communication style, task-oriented communication style, perceived transparency, perceived enjoyment, credibility, and channel engagement. The informativeness factor does not affect channel engagement nor channel loyalty.

This research provided theoretical and practical contributions and implications. This study used different antecedents of communication-style factors compared to previous studies, thus enriching the literature on the factors that influence loyalty channels. Moreover, this study found that product type moderates the relationship between communication style and hedonic and utilitarian motivation. Practically, this study suggested that YouTubers should determine which factors influence audience engagement with and loyalty to their channels. It is necessary to choose the right communication style when presenting a video review on YouTube. The right communication style will increase the audience's pleasure and understanding of the information conveyed, as well as the credibility of the channel. This study suggested that social-oriented and task-oriented communication styles can influence channel loyalty. Therefore, YouTubers can use the results of this study to improve their performance and increase channel loyalty and popularity.

This research has several limitations. First, this study discussed only one platform—namely, YouTube. Second, only one form of video genre on YouTube was analyzed in this study—namely, product reviews in the form of regular or prerecorded videos. Third, the study respondents were not equally distributed; they were mostly 17–23 years of age (68%) and were mostly elementary/junior high/high school students or the equivalent (77.5%).

Based on the results and the limitations of the study, we suggest several recommendations for future research. Future studies can analyze other platforms, such as Instagram, Facebook, Twitter, and Line, as well as other video genres, such as music channels, movie channels, comedy channels, and news channels. It would also be interesting to further analyze livestreaming videos, because they involve direct communication and interaction between the reviewer and the audience. Based on the data collected, almost 50% of the respondents rarely commented on and shared videos. The tendency of the audience to interact minimally with the channel needs to be discussed further in future research. Moreover, future studies can identify other factors and relationships that have not been analyzed in this study, such as the direct relationship between social/task-oriented communication style and customer engagement or loyalty.

## Declarations

### Author contribution statement

Widia Resti Fitriani: Conceived and designed the experiments; Analyzed and interpreted the data; Wrote the paper.

Arif Budi Mulyono: Performed the experiments; Analyzed and interpreted the data.

Achmad Nizar Hidayanto and Qorib Munajat: Conceived and designed the experiments.

### Funding statement

10.13039/501100006378Universitas Indonesia supports this study under PUTI Q1 grant number NKB-1280/UN2.RST/HKP.05.00/2020, titled “Implementation of Message Framing in E-Commerce Applications Using the Design Science Research Approach.” We also thank the Faculty of Computer Science, Universitas Indonesia, for its support.

### Competing interest statement

The authors declare no conflict of interest.

### Additional information

No additional information is available for this paper.

## Figures and Tables

**Table:** 

Instruments of the study	
Social-Oriented Communication Style	Reviewers give warm greetings
	Reviewers are enthusiastic about providing information
	Reviewers are expressive when giving the review (facial expressions, rich in gestures, and other reactions)
	Reviewers invite viewers to comment, like, and give suggestions
	Reviewers look happy while doing the review
	Reviewers are friendly
Task-Oriented Communication Style	Reviewers can provide a great deal of information about the product in a short time
	Reviewers focus on providing the product review
	Reviewers have made the maximum effort to present the video review (including cinematic effects, complete information, and other efforts)
Perceived Transparency	Explanations given by the reviewers can be easily understood
	Explanations given by the reviewers are not confusing
	Explanations given by the reviewers are not wordy
	Reviewers do not use jargon or terms that are difficult to understand
	I understand what the reviewers are saying
Perceived Enjoyment	I feel happy to see the review
	I find it fun to see the review
	I am interested in seeing the review
Informativeness	I can find out the complete physical form of the product being reviewed
	I can find out the nature and characteristics of the product being reviewed
	I can find out the quality of the product being reviewed
	I can find out the function of the product being reviewed
	I can understand how the product is used/consumed
Credibility	I can trust the information provided by the reviewers
	I feel that the information provided by the reviewers is in accordance with reality
	I feel that the information provided by the reviewers is not biased
	I can rely on the information from the reviewers
Engagement	I ... like/dislike the videos on this channel (*options: very rarely, rarely, sometimes, often, and very often*)
	I ... comment on the videos on this channel (*options: very rarely, rarely, sometimes, often, and very often*)
	I ... share the videos from this channel with others (*options: very rarely, rarely, sometimes, often, and very often*)
	I can be very intense (very deep and concerned) when watching videos on this channel
	I enjoy watching videos on this channel
	I am very focused when watching videos on this channel
Loyalty	I will continue watching videos on this channel
	I will watch this channel for another product-review video
	I will recommend this channel to others
	I will watch videos besides product reviews on this channel
